# Supported Pt Nanoparticles on Mesoporous Titania for Selective Hydrogenation of Phenylacetylene

**DOI:** 10.3389/fchem.2020.581512

**Published:** 2020-11-17

**Authors:** Mingzhen Hu, Lei Jin, Yanliu Dang, Steven L. Suib, Jie He, Ben Liu

**Affiliations:** ^1^Jiangsu Key Laboratory of New Power Batteries, Collaborative Innovation Center of Biomedical Functional Materials, School of Chemistry and Materials Science, Nanjing Normal University, Nanjing, China; ^2^Department of Chemistry, University of Connecticut, Mansfield, CT, United States; ^3^Institute of Materials Science, University of Connecticut, Mansfield, CT, United States

**Keywords:** TiO_2_, Pt nanocatalysts, mesoporous chemistry, selective hydrogenation, phenylacetylene

## Abstract

Semi-hydrogenation of alkynes to alkenes is one of the most important industrial reactions. However, it remains technically challenging to obtain high alkene selectivity especially at a high alkyne conversion because of kinetically favorable over hydrogenation. In this contribution, we show that supported ultrasmall Pt nanoparticles (2.5 nm) on mesoporous TiO_2_ (Pt@mTiO_2_) remarkably improve catalytic performance toward semi-hydrogenation of phenylacetylene. Pt@mTiO_2_ is prepared by co-assembly of Pt and Ti precursors with silica colloidal templates *via* an evaporation-induced self-assembly process, followed by further calcination for thermal decomposition of Pt precursors and crystallization of mTiO_2_ simultaneously. As-resultant Pt@mTiO_2_ discloses a high hydrogenation activity of phenylacetylene, which is 2.5 times higher than that of commercial Pt/C. More interestingly, styrene selectivity over Pt@mTiO_2_ remains 100% in a wide phenylacetylene conversion window (20–75%). The styrene selectivity is >80% even at 100% phenylacetylene conversion while that of the commercial Pt/C is 0%. The remarkable styrene selectivity of the Pt@mTiO_2_ is derived from the weakened styrene adsorption strength on the atop Pt sites as observed by diffuse reflectance infrared Fourier transform spectroscopy with CO as a probe molecule (CO-DRIFTS). Our strategy provides a new avenue for promoting alkyne to alkene transformation in the kinetically unfavorable region through novel catalyst preparation.

## Introduction

Selective hydrogenation of alkynes to alkenes is one of the most industrially important reactions (Yang et al., 1998, 2019; Mastalir et al., 2000; Huang et al., 2007; Studt et al., 2008; Yuan et al., 2009; Liu H. et al., 2012; Sheng et al., 2012; Tüysüz et al., 2013; Zhao et al., 2015, 2019; Xie et al., 2016; Xiao et al., 2017; Zhan et al., 2017). The increase of alkene selectivity not only benefits high quality of the downstream polyene products but greatly improves the alkene yields and profitability (Borodziński and Bond, 2006, 2008; McCue and Anderson, 2015; Hu et al., 2018a). Pt-based catalysts still suffer from low selectivity to alkenes especially in higher alkyne conversion windows, despite its high activity for hydrogenation (Li et al., 2017; Tang et al., 2017; Wang et al., 2018). In theory, the adsorption and activation of alkynes are favored thermodynamically over Pt compared to alkenes. However, as alkynes approach a high conversion, alkenes become kinetically favorable to adsorb on Pt, leading to a full hydrogenation to alkanes and thus a sharp decrease in alkene selectivity (Yang et al., 2012; McCue and Anderson, 2015). For example, commercial Pd/C or Pt/C catalysts generally produce alkane at 100% alkyne conversion (Li et al., 2012). The lack of control of alkene selectivity at higher alkyne conversions greatly limits alkene yields. As a result, selective hydrogenation of alkynes is generally conducted at low alkyne conversions to generate enhanced yields of alkenes.

The key to solving this problem lies in developing more efficient Pt-group metal catalysts with weakened alkene adsorption strength, which facilitates the alkenes desorption rather than over hydrogenation to alkanes. Compared with strong σ-bonded alkenes, π-bonded alkenes are more favorable to desorb from the active Pt-group metal (Wan and Zhao, 2007; Armbrüster et al., 2010; Mitsudome et al., 2016; Feng et al., 2017). For example, Li et al. found that the π-bonded ethylene was 0.75 eV more facile than the σ-bonded ethylene to desorb from Pd surfaces based on density-functional theory (DFT) calculations (Feng et al., 2017). This in turn greatly favors high ethylene selectivity where 80% ethylene selectivity is achieved, even when acetylene is fully converted. The π-mode alkene adsorption is mainly on atop metal sites (Pei et al., 2017). To improve the alkene selectivity in selective hydrogenation of alkyne, both nanoparticles (NPs) downsizing and alloying strategies have been carried out to enrich the atop sites of Pt-group metal catalysts (Hu et al., 2018b). On the other hand, metal-support interaction has long been employed to tune catalytic properties of catalysts especially the small NPs with strong interactions with supports (Teschner et al., 2008). A reduced hydrogen reduction temperature is generally observed on the small NPs functionalized supports, which greatly benefits hydrogenation activity by virtue of strong hydrogen spillover (Wang et al., 2015; Wei et al., 2018). The decrease of particle sizes also enables more surface-exposed and low-coordinated active sites in the form of corner or edge sites, potentially favoring weak π-adsorbed alkenes (Kleis et al., 2011; Yang et al., 2013; Wu et al., 2019). Thus, smaller Pt NPs hold great potential to catalyze semi-hydrogenation of alkynes with high catalytic activity and alkene selectivity.

**Figure 1:**
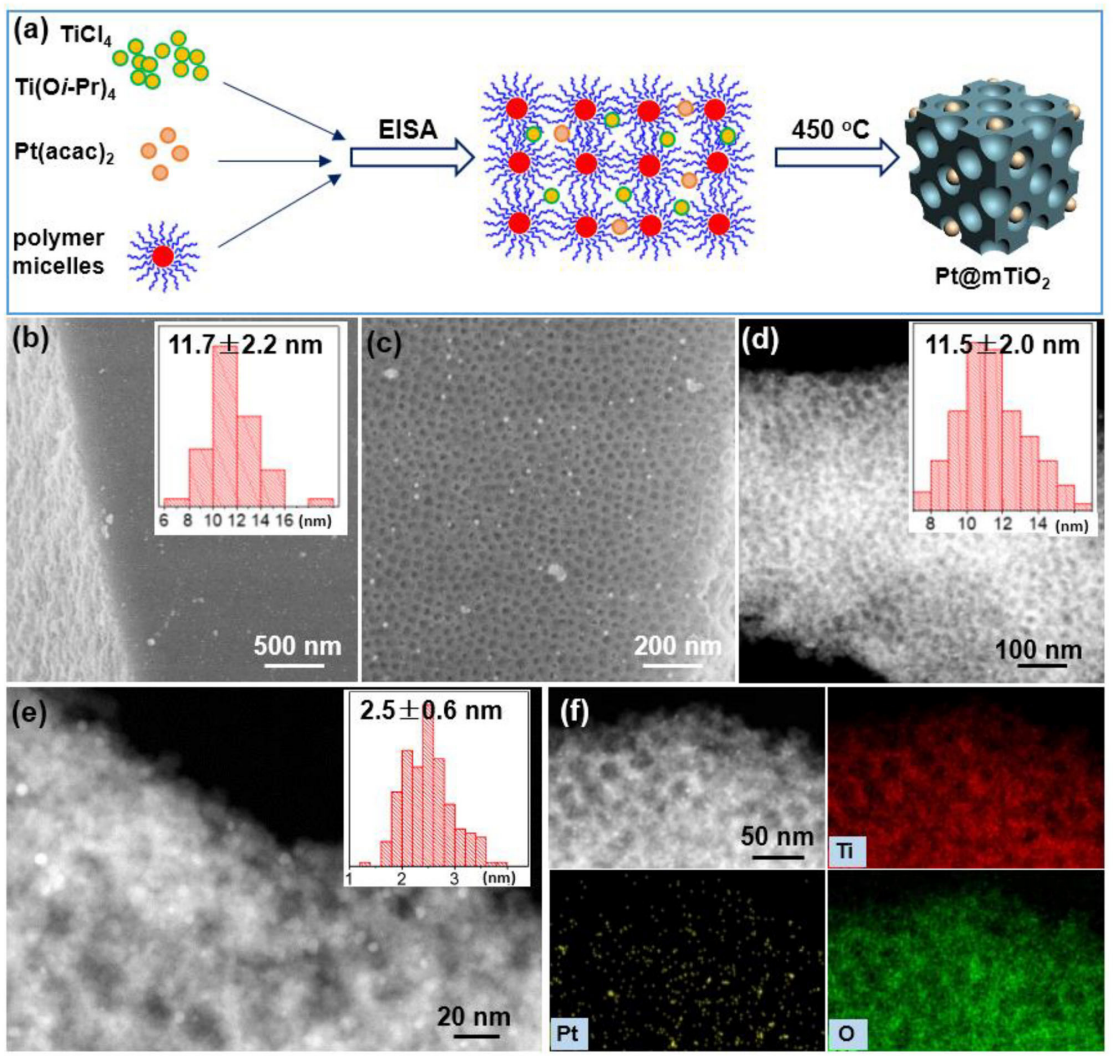
**(a)** Schematic illustration of preparing 2.5 nm Pt NPs supported within the mesoporous TiO_2_. **(b,c)** SEM images, **(d,e)** HAADF-STEM images, and **(f)** EDS mapping images of Pt@mTiO_2_. The inset of **(b,d,e)** are size/diameter distributions of mesopores from SEM, mesopores from TEM, and Pt NPs from STEM.

In the current study, we present a highly selective catalyst that contains ultrasmall Pt NPs (2.5 nm), strong Pt-support (Pt-TiO_2_) interaction, and high surface area (mesoporous framework) for selective hydrogenation of phenylacetylene to styrene. Supporting ultrasmall Pt NPs on mesoporous TiO_2_ (Pt@mTiO_2_) were prepared using an evaporation-induced self-assembly (EISA) method (Figure 1a). For this synthesis, platinum acetylacetonate (Pt(acac)_2_) was mixed with titanium sources and structure-directing polymers in EISA (Liu X. et al., 2012). Upon annealing, ultrasmall Pt NPs were synthesized and encapsulated within the ordered mTiO_2_ support. The ordered mesopores of mTiO_2_ provide a powerful nanoconfinement and endow the ultrasmall Pt nanoparticles with excellent size uniformity and thermal stability. The monodisperse Pt NPs with sizes of around 2.5 nm remained stable after calcination at 450°C. Pt NPs supported on mTiO_2_ (Pt@TiO_2_) exhibited excellent catalytic properties toward selective hydrogenation of phenylacetylene. The selectivity to styrene remained at 100% in the phenylacetylene conversion range of 20–75%. Even at 100% phenylacetylene conversion, Pt@TiO_2_ exhibited >80% styrene selectivity. The abundant surface atop Pt sites of the Pt@mTiO_2_ accounted for the high styrene selectivity as revealed by CO-DRIFTS. In addition, the specific mass activity of the 2.5 nm Pt@mTiO_2_ reached as high as 0.5 mol h^−1^ per gram of Pt metal, which is 2.5 times higher than that of the commercial Pt/C catalyst (0.2 mol h^−1^ per gram of Pt metal).

## Experimental Section

### Chemicals and Materials

Titanium chloride (TiCl_4_), titanium tetraisopropoxide (TIPO), 1,4-dioxane, phenylacetylene, commercial Pt/C (5 wt%) and platinum acetylacetonate (Pt(acac)_2_) were purchased from Sigma-Aldrich. The CDCl_3_ was purchased from Cambridge Isotope Laboratories, Inc. All the chemicals were used without further purification unless otherwise noted. Silane-containing block copolymers of poly(ethylene oxide)-*block*-poly(3-(trimethoxysilyl)propyl methacrylate) (PEO-*b*-PTMSPMA, M_n_ = 56.9 kg mol^−1^) was prepared through atom transfer radical polymerization (ATPR) method as described in our previous reports (Tauster et al., 1978; Wang et al., 2015; Zhang et al., 2015; Feng et al., 2017; Hu et al., 2020). The polymer micelles were prepared in water/ethanol mixtures and dialyzed in ethanol prior to use. Deionized water (High-Q, Inc. 103S Stills) with a resistivity of >10.0 MΩ was used in all experiments.

### Synthesis of Pt@mTiO_2_

Pt@mTiO_2_ was prepared using an EISA method. In a typical synthesis, 400 μL of newly prepared TiCl_4_ solution (10 % in ethanol, volume ratio) was added to 5.0 mL of PEO-*b*-PTMSPMA micelles solution (15.0 mg mL^−1^ in ethanol) under stirring. Two hundred and seventy micro liter of TIPO was then added into the above solution, followed by mild stirring for 30 min. 2.0 mg of Pt(acac)_2_ dissolved in 1.0 mL of ethanol was added dropwise where the theoretical mole ratio of Pt to Ti is 0.004 (corresponding to 1.0 wt% Pt loading in the obtained Pt@mTiO_2_). After sufficient stirring for 30 min, the mixture solution was transferred into a Petri dish to evaporate the solvent at 40°C for 24 h and 100°C for another 12 h. After this procedure, the obtained intermediates were allowed for calcination at 450°C under air for 2 h. A template removal process is further carried out by washing with hot 2.0 M NaOH solution for 30 min at 50°C. The Pt loading given by EDS measurements is 1 wt%, agreeing well with the initial Pt feed ratio. Through subsequent drying in vacuum at 50°C overnight, Pt@mTiO_2_ was finally obtained. About 3.6 nm Pt NPs on mesoporous TiO_2_ is also prepared as a control using similar procedures except that the thermal calcination is carried out at 650°C for 2 h.

### Selective Hydrogenation of Phenylacetylene Catalytic Evaluations

Selective hydrogenation of phenylacetylene over the Pt@mTiO_2_ catalysts was carried out by the following procedures. Firstly, 10.0 mg of catalyst was added to 3.0 mL of 1,4-dioxane in a 15 mL processed glass bottle. After 20 min sonication, 100.0 mg (1.0 mmol) of phenylacetylene was further added to the homogeneous suspension and stirred for another 30 min. Before transferring the processed bottle to a 25 mL high-pressure stainless-steel reactor, the magnet is taken out, leaving the reaction happen at static conditions without stirring. After purging with hydrogen several times, the reactor was pressurized with 50 psi (pounds per square inch) hydrogen and reacted for varied times. After the reaction finished, the whole reactor was cooled down in an ice bath. The postreaction suspension was separated by centrifugation at 8,000 rpm. The resultant product was kept in a 5 mL test tube and the proton nuclear magnetic resonance (^1^H NMR) analysis was carried out using CDCl_3_ as solvent to calculate the conversion of phenylacetylene and the selectivity to styrene. The reaction kinetics were measured both for the Pt@mTiO_2_ and commercial Pt/C catalysts. The specific reaction rate was determined as the consumption rate of the phenylacetylene (normalized to the mass of Pt metal per hour) by controlling the phenylacetylene conversion in the kinetic region. The specific rate in this work was calculated using the following equation:

(1) $$\begin{array}{r} r=\frac{N}{w*t}\; \end{array}$$

where r is the specific rate, N is the converted phenylacetylene amount in mole, w is supported metal loading weight (gram), and t is the reaction (minute).

The turnover frequency (TOF) is calculated based on the following equation:

(2) $$\begin{array}{r} TOF=\frac{N}{\left(w/M\right)*D*t}\; \end{array}$$

where N is the converted phenylacetylene amount in mole, w is supported Pt loading weight (gram), M is the molar mass of Pt, D is metal dispersion of Pt and t is the reaction (second). The metal dispersion is calculated according to previous reports (Dominguez-Dominguez et al., 2008; Qiao et al., 2011).

The stability tests of the Pt@mTiO_2_ and the commercial Pt/C were carried out by recycling the catalyst for 5 consecutive cycles.

### Characterization

Scanning electron microscopy (SEM) was conducted on a FEI Nova NanoSEM 450. Bright and dark-field transmission electron microscopy (TEM) was performed on JEM-2100F FETEM. The X-ray diffraction (XRD) patterns were collected on a Bruker D2 Phaser. The small angle X-ray scattering (SAXS) patterns were recorded on a Bruker Nano STAR instrument. X-ray photoelectron spectroscopy (XPS) analysis was conducted on ESCALAB 250 Xi X-ray photoelectron spectrometer with Al Kα radiation. The N_2_ sorption measurements were conducted on a Micromeritics ASAP 2020 Surface Area and Porosity Analyzer. ^1^H NMR spectroscopy was collected on a Bruker Avance 300 MHz spectrometer. Diffuse reflectance Fourier transform infrared (DRIFT) spectroscopy was carried out using a Nicolet IS50 spectrometer using CO as a probe molecule. The DRIFT cell was filled with 20 mg of catalysts and was treated at 100°C with nitrogen before CO was introduced.

## Results and Discussion

Mesoscopic and microscopic structures of the Pt@mTiO_2_ were thoroughly characterized with SEM and high-angle annular dark field scanning TEM (HAADF-STEM) (Figure 1). The SEM image in Figure 1b shows the mesopores throughout entire mTiO_2_ support. Supplementary Figure 1 shows that mTiO_2_ displays bulk cubic morphology with a broad side length distribution. The zoomed-in SEM picture in Figure 1c confirms the uniform distribution of mesopores. The average pore size of mTiO_2_ is 11.7 ± 2.2 nm as given in the inset of Figure 1b. Since Pt NPs are small, there is no obvious contrast on bright field TEM. The HAADF-STEM technique is further used to identify the ultrasmall Pt NPs. The HAADF-STEM images of Pt@mTiO_2_ in Figures 1d–f reveal the well-dispersed Pt NPs. Pt NPs with a higher electron density are brighter under dark-field STEM image while TiO_2_ is darker. The average size of Pt NPs is 2.5 ± 0.5 nm as plotted in Figure 1e. The pore size of mTiO_2_ is about 11 nm from HAADF-STEM characterization, which agrees well with the above SEM observations. Figure 1f shows energy dispersive X-ray spectroscopy (EDS) mapping images of Pt@mTiO_2_, further confirming fine dispersion of these 2.5 nm Pt NPs in mTiO_2_ support. The Pt loading of Pt@mTiO_2_ is further determined by EDS measurements. As show in Supplementary Figure 2 and Supplementary Table 1, the Pt loadings obtained by EDS measurements of Pt@mTiO_2_ are about 1 wt%, agreeing well with the initial feed ratio (1 wt%). This observation implies no loss of Pt during preparation processes. By comparison, we have also carried out TEM observations of the commercial Pt/C catalyst. As displayed in Supplementary Figure 3, the Pt NPs in commercial Pt/C show a very wide size distribution averaging about 3 nm as reported previously (Cui et al., 2019). The Pt loading of the commercial Pt/C is 5 wt% as received.

**Figure 2:**
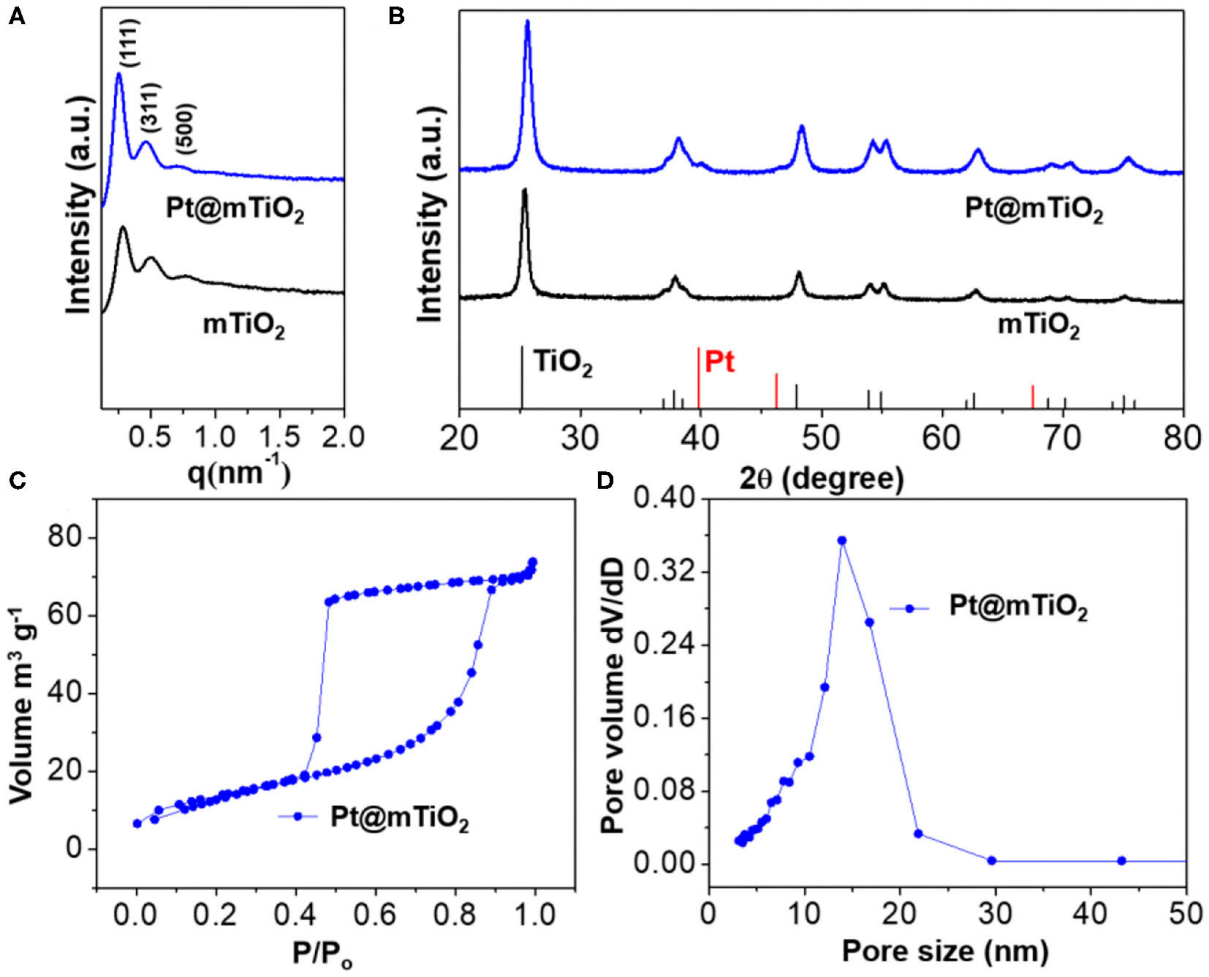
**(A)** SAXS, and **(B)** wide-angle XRD of Pt@mTiO_2_ and pure mTiO_2_. **(C)** N_2_ adsorption-desorption isotherms, and **(D)** pore size distributions of Pt@mTiO_2_.

To examine the mesoporous structures and crystallographic information of the Pt@mTiO_2_, we carried out small-angle X-ray scattering (SAXS) and wide-angle X-ray diffraction (XRD) characterization. Figure 2A is the SAXS patterns of the Pt@mTiO_2_ and the pure mTiO_2_ support. These data show a set of strong scattering peaks that are clearly identified and assigned to (111), (311), and (500) reflections of the face-centered cubic (fcc) mesostructure. The average cell parameters were calculated to be 38 nm as displayed in Supplementary Table 2. The SAXS signals are almost the same for Pt@mTiO_2_ and mTiO_2_, indicating well-defined mesoporous structures. Figure 2B displayed the wide-angle XRD patterns, suggesting the anatase TiO_2_ phase for both Pt@mTiO_2_ and mTiO_2_ (JCPDS 00-064-0863). Unfortunately, due to the ultrasmall size, metallic Pt NPs (JCPDS 04-0802) only showed very broad and weak XRD diffraction (Xiang et al., 2011; Prinz et al., 2014). Benefiting from the well-defined mesoporous structures, the BET specific surface area of the Pt@TiO_2_ is 42 m^2^g^−1^ with an average of pore size of 14 nm as displayed in Figures 2C,D.

**Figure 3:**
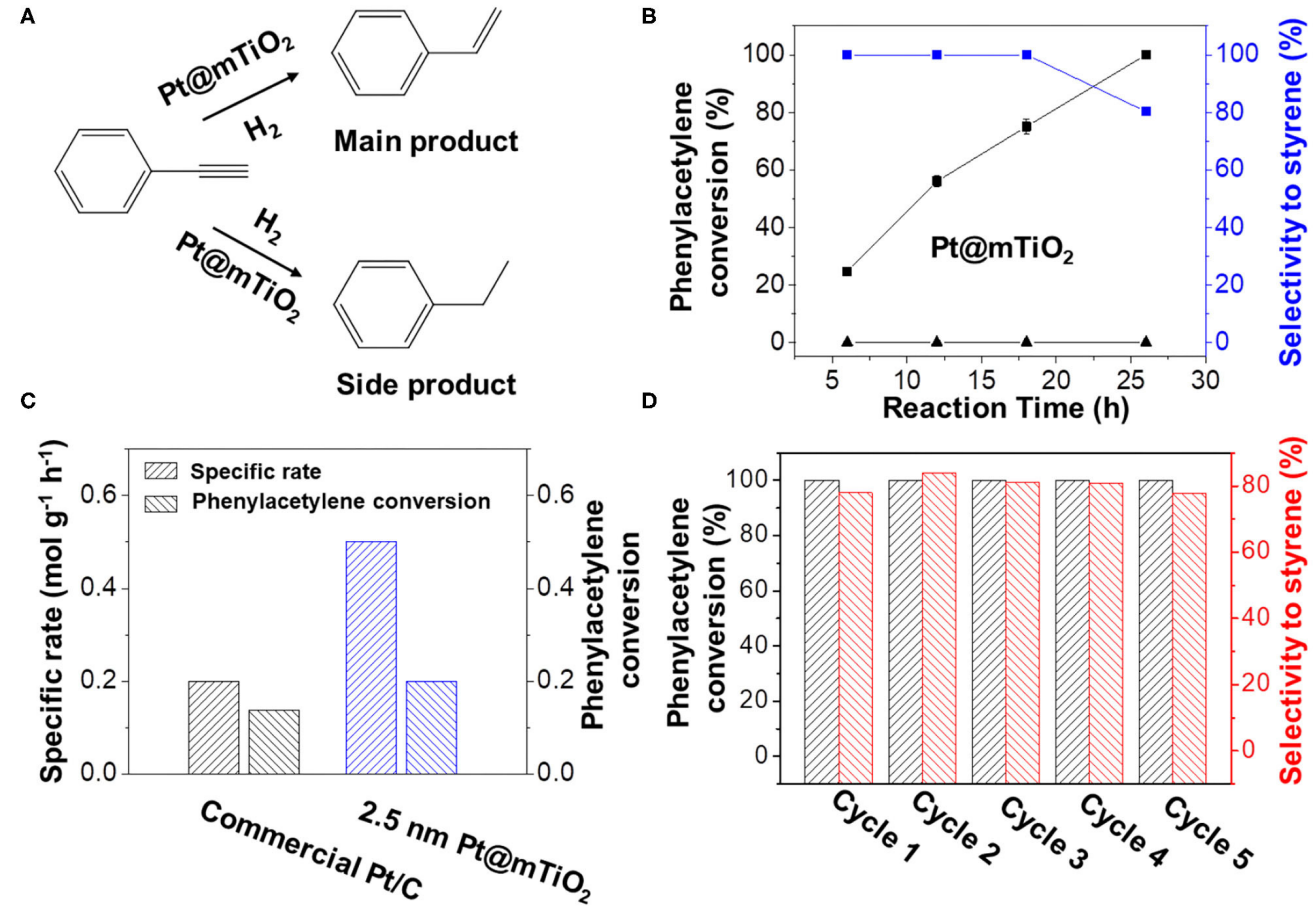
**(A)** Hydrogenation of phenylacetylene over Pt@mTiO_2_ and commercial Pt/C. **(B)** Phenylacetylene conversion and styrene selectivity of Pt@mTiO_2_ as a function of reaction time. The bottom line shows that the mTiO_2_ is inert for converting phenylacetylene. **(C)** Specific mass activity of the Pt@mTiO_2_ and the commercial Pt/C catalyst. **(D)** Catalytic stability measurements of the Pt@mTiO_2_ by recovering the catalysts for five consecutive cycles. Reaction conditions: 10 mg of catalyst is dispersed in 3 mL of 1,4-dioxane that contains 0.1 g of phenylacetylene at 50°C and 50 psi of hydrogen for different reaction times.

Pt@mTiO_2_ has multiple advantageous properties, including ultrasmall Pt NPs and mesoporous framework with high surface area, which possibly benefit the selective hydrogenation of alkynes to alkenes. In this manuscript, selective hydrogenation of phenylacetylene was chosen as a probe reaction since the semi-hydrogenation product of styrene is industrially important (Figure 3A). As a control, commercial Pt/C was also investigated. Pt@mTiO_2_ was favorable for catalyzing the hydrogenation of phenylacetylene to styrene, while commercial Pt/C fully hydrogenated phenylacetylene to ethylbenzene. Figure 3B showed the phenylacetylene conversion and the styrene selectivity over Pt@mTiO_2_ as a function of reaction time. Phenylacetylene was selectively converted to styrene in a wide phenylacetylene conversion window (20–75%), only when phenylacetylene reached full conversion, styrene selectivity slightly dropped to 80.3%. This result (80.3% styrene selectivity at 100% phenylacetylene conversion) is among the best reported to date as demonstrated in Supplementary Table 3 (Zhao et al., 1998; Kleis et al., 2011; Behrens et al., 2012; Montesano Lopez, 2014; Alig et al., 2019; Han et al., 2019). By comparison, pure mTiO_2_ is inactive and shows no activity to convert phenylacetylene in the reaction time range from 7 to 26 h (Supplementary Figure 4). We have also compared the catalytic properties of Pt@mTiO_2_ with commercial Pt/C catalysts. Phenylacetylene was fully over hydrogenated to ethylbenzene in the measuring time range from 7 to 26 h with a 100% ethylbenzene selectivity as shown in Supplementary Figure 5.

Figure 3C shows the catalytic rates of Pt@mTiO_2_ and commercial Pt/C normalized to the Pt mass per unit time. By controlling the phenylacetylene conversion in the kinetic region (10–20% of conversion), the rate characterizes the intrinsic activity of the catalyst. The catalytic rate of Pt@mTiO_2_ is 0.5 mol g_Pt_^−1^ h^−1^, 2.5 times higher than that of commercial Pt/C (0.2 mol g_Pt_^−1^ h^−1^). In addition, the TOF value of the Pt@mTiO_2_ is calculated to be 4.1 s^−1^, which is more than 2 times that of the commercial Pt/C (2.0 s^−1^), demonstrating a higher intrinsic activity. According to previous results, the particle size of Pt NPs is intimately connected with the amount of surface-exposed active sites, which in turn affects the activity (Zhang et al., 2007; Qiao et al., 2011). The smaller sized Pt in Pt@mTiO_2_ results in more exposed surface Pt sites compared with commercial Pt/C (average size of 3 nm), which benefits high catalytic activity. Catalytic stability is another concern for practical applications. To test the catalytic stability of Pt@mTiO_2_, we have carried out recycling experiments of the post-reacted Pt@mTiO_2_ at 50°C and 50 psi hydrogen. As shown in Figure 3D, styrene selectivity remains around 80% over the Pt@mTiO_2_ while phenylacetylene conversion remains at 100% even after five catalytic recycles, showing excellent catalytic stability. By comparison, styrene selectivity over the commercial Pt/C is 0% for the five consecutive measurements at 100% phenylacetylene conversion as displayed in Supplementary Figure 6. By virtue of the nanoconfinment enabled by mesoporous mTiO_2_ supports, the average particle size of the post-reacted Pt@mTiO_2_ remains the same without any growth or aggregation as displayed in Supplementary Figure 7.

**Figure 4:**
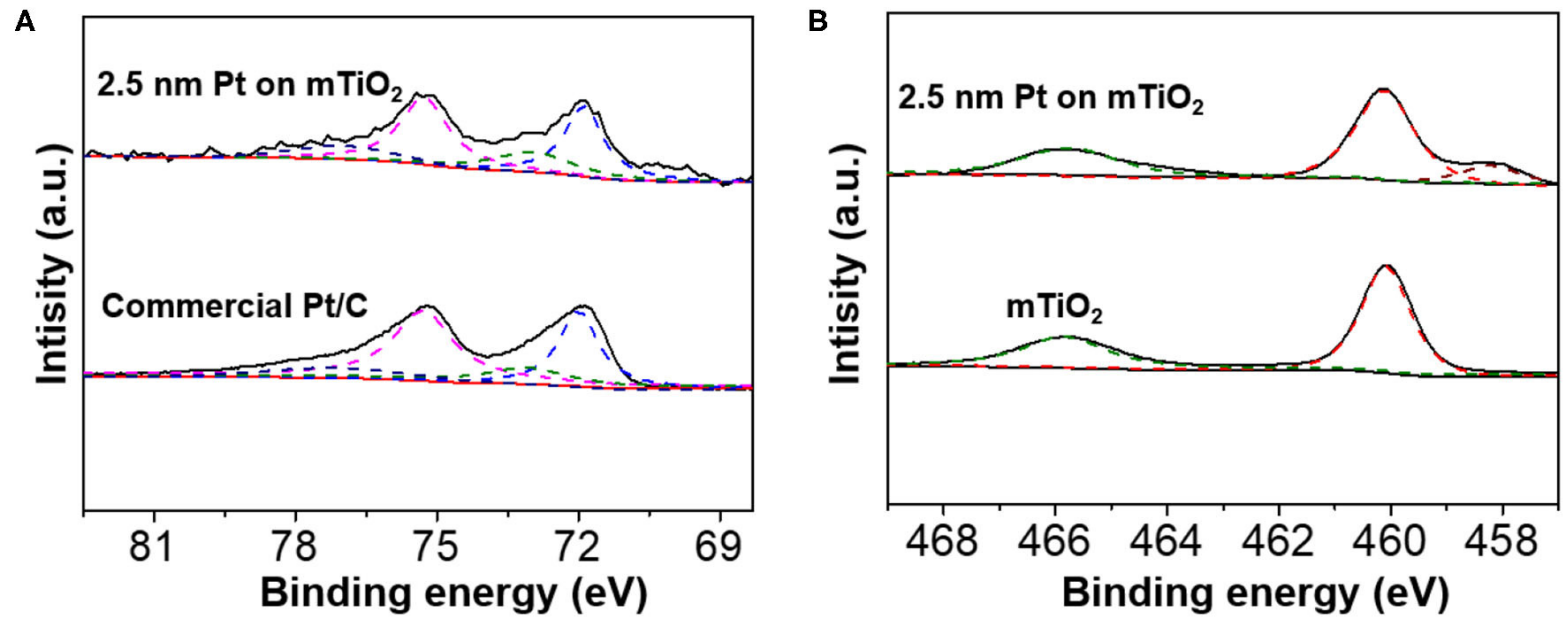
**(A)** High-resolution Pt 4f XPS spectra of the 2.5 nm Pt NPs on mTiO_2_ and commercial Pt/C. **(B)** High-resolution Ti 2p XPS spectra of the 2.5 nm Pt NPs on mTiO_2_, and pure mTiO_2_.

To reveal the intrinsic nature why Pt@mTiO_2_ presented excellent catalytic properties for the selective hydrogenation of phenylacetylene, the surface properties of Pt were carefully studied. We first carried out X-ray photoelectron spectroscopy (XPS) for Pt@mTiO_2_. Figure 4A is the high-resolution Pt 4f XPS spectra of Pt@mTiO_2_ and commercial Pt/C. The well-resolved XPS binding energy peaks centered at 71.9 and 75.3 eV of Pt@mTiO_2_ are ascribed to metallic Pt 4f_7/2_ and Pt 4f_5/2_, respectively. For the commercial Pt/C, the Pt 4f_7/2_ and Pt 4f_5/2_ shifted to a slightly higher binding energy at 72 and 75.4 eV, respectively. The shift of Pt 4f peaks is indicative of the surface electron density difference of the two samples. 2.5 nm Pt NPs is slightly electron-rich compared to that of the commercial Pt/C. The electron-rich 2.5 nm Pt NPs are more effective to transfer electrons to the π^*^ molecular orbitals of phenylacetylene, which in turn promotes phenylacetylene activation and improves catalytic reactivity (Wan and Zhao, 2007). Both the Pt@mTiO_2_ and commercial Pt/C show existence of Pt^2+^ species as identified at around 73.1 and 77.1 eV, respectively, which may derive from surface oxidation (Miller et al., 2014). The Pt^2+^/Pt^0^ ratio, as measured from the integration areas in the fitting cure, is 0.43 for 2.5 nm Pt NPs. By comparison, this is much lower for commercial Pt/C with a Pt^2+^/Pt^0^ ratio of 0.35. This result implies that Pt is more surface oxidized in 2.5 nm Pt NPs, which is further evidenced in Ti 2p XPS spectra. As shown in Figure 4B, a strong Ti^3+^ XPS binding energy peak at around 458.2 eV is found in 2.5 nm Pt NPs on mTiO_2_, which is absent in the pure mTiO_2_ (Yang et al., 2017; Ou et al., 2018). These results show that 2.5 nm Pt NPs have a stronger interaction with the mTiO_2_ support where Pt NPs are partially oxidized and demonstrate electron back-donation to reduce Ti^4+^ to Ti^3+^ of mTiO_2_. The stronger metal-support interaction is of great importance in stabilizing small NPs as revealed by a series of reports (Lu and Schüth, 2006). In the current case, the excellent thermal stability of 2.5 nm Pt NPs supported on mTiO_2_ may also derive from strong metal-support interactions.

**Figure 5:**
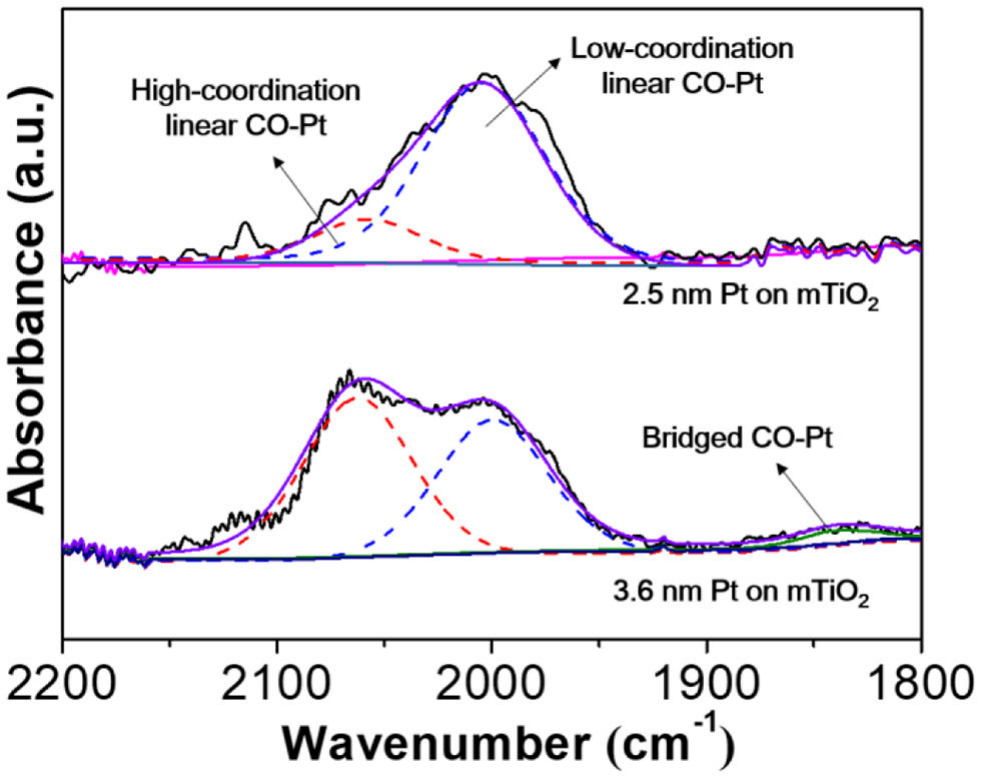
Diffuse reflectance infrared Fourier transform spectroscopy with CO molecules of the 2.5 nm Pt NPs on mTiO_2_ and the 3.6 nm Pt NPs on mTiO_2_.

Small NPs afford abundant surface low-coordinated corner or edge sites, which potentially modifies reactant/product adsorption modes that are quite different from that on larger ones (Zelcer and Soler-Illia, 2013). For example, alkenes prefer a weak π adsorption on low-coordinated Pt sites such as corner, edge, or single atom sites of small Pt NPs rather than a strong σ adsorption on continuous Pt lattices of larger Pt NPs. The weakening of alkene adsorption strength greatly favors high alkene selectivity (Yang et al., 2013; Zhang et al., 2019). To this end, CO-DRIFTS was further carried out to understand the high selectivity to styrene on Pt@mTiO_2_. For the commercial Pt/C, it is very difficult to collect CO-DRIFT spectra because of signal blocking of carbon supports. Thus, we further prepared 3.6 nm Pt supported on mTiO_2_ by calcination at a higher annealing temperature (650°C), which preserved the mesoporous structure but with slightly larger Pt NPs (3.6 nm) (Supplementary Figure 8). Supplementary Figure 9 shows that both the catalytic activity and styrene selectivity of the 3.6 nm Pt NPs on mTiO_2_ are much lower than that of the 2.5 nm Pt NPs on mTiO_2_. Figure 5 shows the CO-DRIFTS curve of 2.5 nm Pt NPs and 3.6 nm Pt NPs on mTiO_2_, respectively. The CO adsorption models on 2.5 nm Pt NPs are quite different from that on 3.6 nm Pt NPs. The fitted results show that only the linear CO adsorption mode is observed on the 2.5 nm Pt NPs on mTiO_2_ while the 3.6 nm Pt NPs on mTiO_2_ display both linear CO adsorption and bridged CO adsorption modes in the wavenumber window between 1,800 and 2,200 cm^−1^ (Singh et al., 2018). The linear CO adsorption of both 2.5 nm Pt NPs on mTiO_2_ and 3.6 nm Pt NPs on mTiO_2_ are related to the surface atop Pt sites (Wieckowski, 1999). Due to the Pt coordination differences, the linear CO adsorption bands of the 2.5 nm Pt NPs on mTiO_2_ and the 3.6 nm Pt NPs on mTiO_2_ are divided into two regions that are referred to as the low-coordination Pt-CO linear adsorption band and high-coordination Pt-CO linear adsorption band centered at around 2,004 and 2,160 cm^−1^, respectively (Redina et al., 2015; Singh et al., 2018). The low-coordination Pt-CO linear adsorption to high-coordination Pt-CO linear adsorption ratio of 2.5 nm Pt NPs on mTiO_2_ is calculated to be 4.8, which is 6 times higher than that of 3.6 nm Pt NPs on mTiO_2_. This result demonstrates high unsaturated coordination of the Pt sites in 2.5 nm Pt NPs on mTiO_2_ due to the smaller Pt NPs size as compared with 3.6 nm Pt NPs on mTiO_2_, which agrees well with the above HAADF-STEM observations. The schematic models of Pt NPs with diameters of 2.5 and 3.6 nm are also constructed, respectively, as displayed in Supplementary Figure 10. Smaller Pt NPs show more abundant surface corner and edge sites, which accounts for the pure linear CO adsorption revealed via CO-DRIFTS measurements. The unique surface structures of the 2.5 nm Pt NPs on mTiO_2_ greatly benefit weak styrene adsorption in selective hydrogenation of phenylacetylene through a π adsorption mode, which favors styrene desorption and high styrene selectivity.

## Conclusion

To summarize, we report the preparation of mesoporous TiO_2_ confined ultrasmall Pt nanoparticles with an average particle size of 2.5 ± 0.5 nm using an EISA method. As-resulted Pt@mTiO_2_ displayed excellent catalytic activity and styrene selectivity in semi-hydrogenation of phenylacetylene. The hydrogenation activity of Pt@mTiO_2_ displays a 2.5-fold increase over that of the commercial Pt/C. A 100% styrene selectivity is also obtained over the Pt@mTiO_2_ in a wide phenylacetylene conversion range of 20–75%. The styrene selectivity is remarkably above 80% even at full phenylacetylene conversion. The excellent styrene selectivity is ascribed to the abundant surface atop Pt sites as given by the CO-DRIFTS. In addition, the Pt@mTiO_2_ also shows excellent catalytic stability as demonstrated in five consecutive recycling experiments, which shows great potential for practical applications. Our work is expected to open up new opportunities for developing highly efficient alkene-to-alkene conversion catalysts by novel synthesis.

## Data Availability Statement

The original contributions presented in the study are included in the article/Supplementary Material, further inquiries can be directed to the corresponding author.

## Author Contributions

MH, SS, JH, and BL conceived the projects. MH carried out the experiments and analyzed data in the assist of LJ. YD did high-resolution TEM and analyzed the data. All authors co-wrote the manuscript.

## Conflict of Interest

The authors declare that the research was conducted in the absence of any commercial or financial relationships that could be construed as a potential conflict of interest.
