# Supplementary material for: Supported Pt Nanoparticles on Mesoporous Titania for Selective Hydrogenation of Phenylacetylene
Source: Front Chem. 2020 Nov 17;8:581512. doi: 10.3389/fchem.2020.581512 (PMC7718006; doi:10.3389/fchem.2020.581512)
Supplement: Supplementary file 1 [file Data_Sheet_1.docx]

**Supported Pt Nanoparticles on Mesoporous Titania for Selective Hydrogenation of Phenylacetylene**

Mingzhen Hu,^1,2^ Lei Jin,^2^ Yanliu Dang,^3^ Steven L. Suib,^2,3^ Jie He^2,3^ and Ben Liu^1,*^

^1^Jiangsu Key Laboratory of New Power Batteries, Collaborative Innovation Center of Biomedical Functional Materials, School of Chemistry and Materials Science, Nanjing Normal University, Nanjing 210023, China; ^2^Department of Chemistry, and ^3^Institute of Materials Science, University of Connecticut, Storrs, Connecticut 06269, United States.

*Email: ben.liu@njnu.edu.cn

**Figure Contents**

Figure S1. SEM image of the mTiO_2_ at low magnification.

Figure S2. EDS elementary loading measurement of the Pt@mTiO_2_.

Figure S3. TEM images of the commercial Pt/C catalyst.

Figure S4. Phenylacetylene conversion over the mTiO_2_ from 7 h to 26 h.

Figure S5. Phenylacetylene conversion and styrene selectivity of commercial Pt/C as a function of reaction time from 7 h to 26 h.

Figure S6. Catalytic stability test of the commercial Pt/C.

Figure S7. HAADF-STEM image of the postreaction and Pt@mTiO_2_ after five catalytic recycling measurement.

Figure S8. HAADF-STEM images and size distributions of the 3.6 nm Pt NPs on mTiO_2_.

Figure S9. Phenylacetylene conversion and styrene selectivity of the 3.6 nm Pt NPs on mTiO_2_ as a function of reaction time.

Figure S10. Schematic model of 2.5 nm Pt NPs and 3.6 nm Pt NPs.

**Table Comments**

Table S1. Comparisons of initial Pt loadings and that from EDS measurements of the Pt@mTiO_2_.

Table S2. Cell parameters of the Pt@TiO_2_ and mTiO_2_.

Table S3. Styrene selectivity at full phenylacetylene conversion of Pt@mTiO_2_ and previous results.


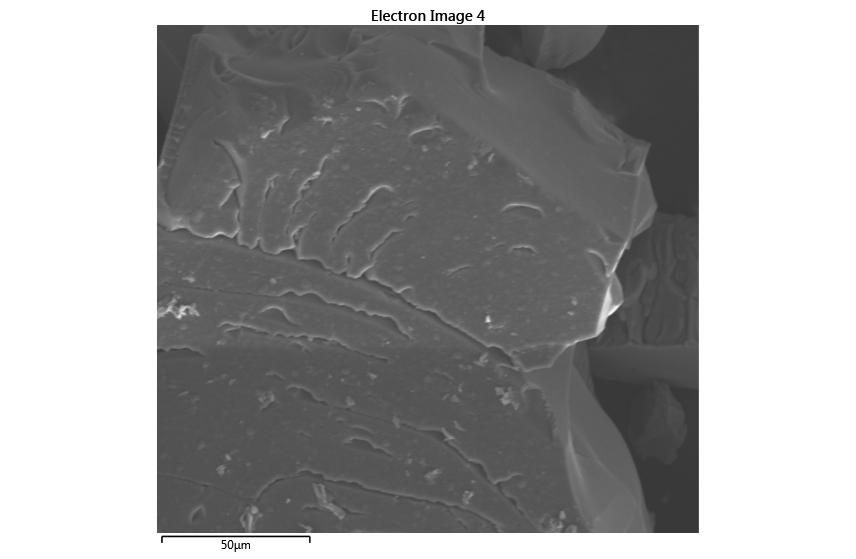


**Figure S1.** SEM image of the mTiO_2_ at low magnification.


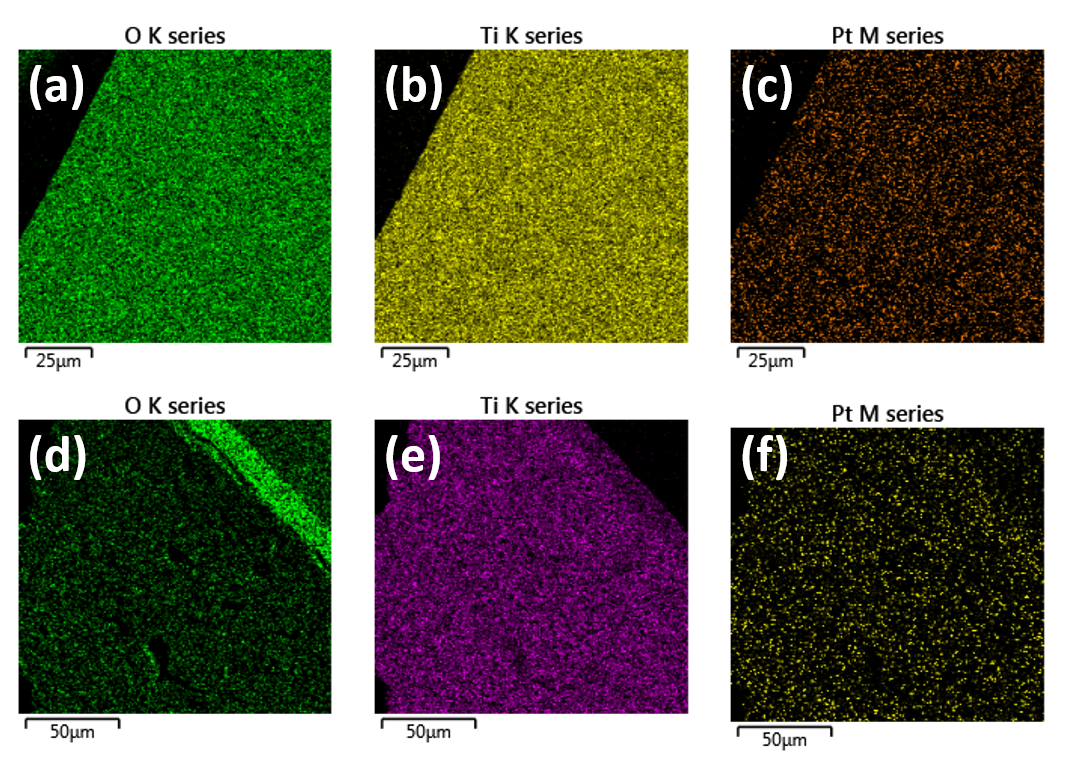


**Figure S2.** (a-c) SEM EDS elemental mappings of the Pt@mTiO_2_.


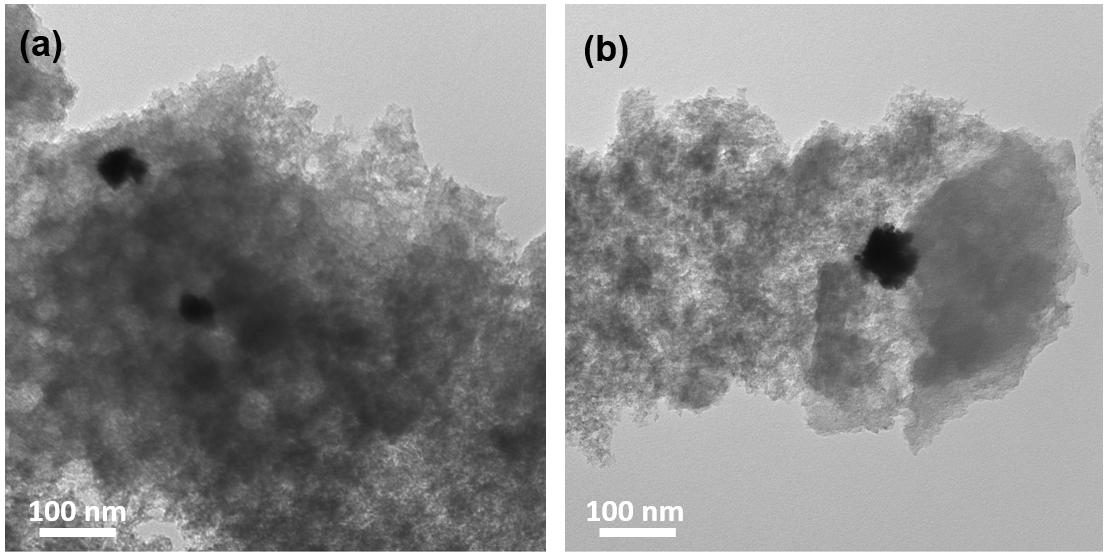


**Figure S3.** (a,b) TEM images of the commercial Pt/C catalyst.


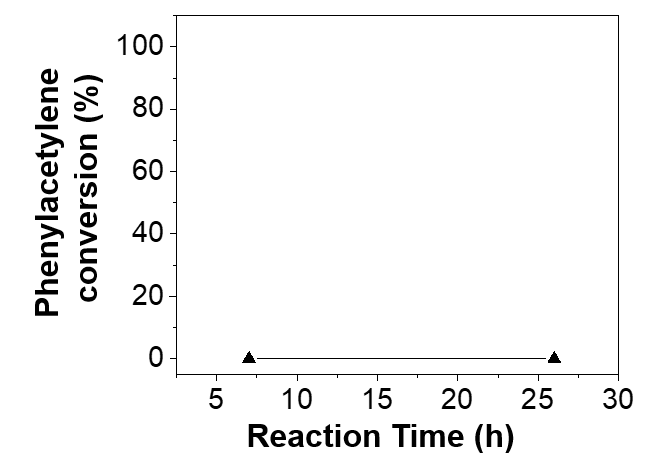


**Figure S4.** Phenylacetylene conversion over the mTiO_2_ from 7 h to 26 h.

Reaction conditions: 10 mg of mTiO_2_ was dispersed in 3 mL of 1,4-dioxane with 0.1 g of phenylacetylene at 50 °C and 50 psi of hydrogen for different reaction times.


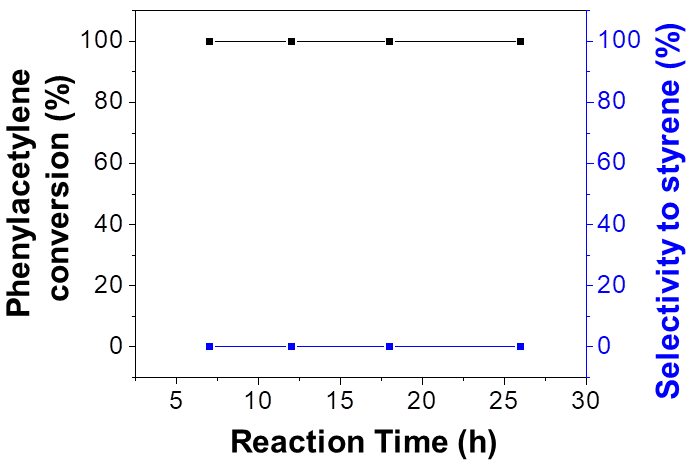


**Figure S5.** Phenylacetylene conversion over the commercial Pt/C from 7 h to 26 h.

Reaction conditions: 10 mg of mTiO_2_ was dispersed in 3 mL of 1,4-dioxane with 0.1 g of phenylacetylene at 50 °C and 50 psi of hydrogen for different reaction times.


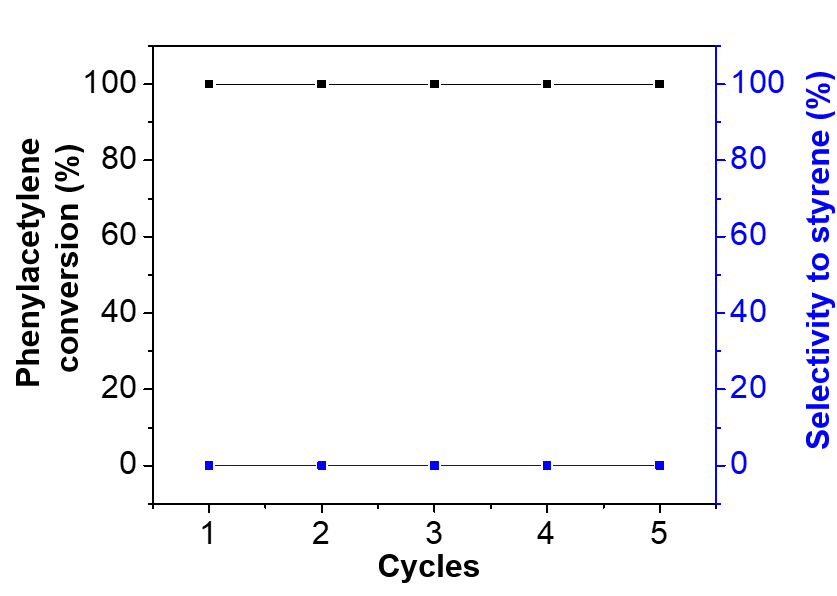


**Figure S6.** Phenylacetylene conversion and styrene selectivity of the commercial Pt/C after five catalytic cycling measurement.

Reaction conditions: 10 mg of commercial Pt/C was dispersed in 3 mL of 1,4-dioxane with 0.1 g of phenylacetylene at 50 °C and 50 psi of hydrogen for 26 hours.





**Figure S7.** HAADF-STEM images of the postreaction and Pt@mTiO_2_ after five catalytic recycling measurement.


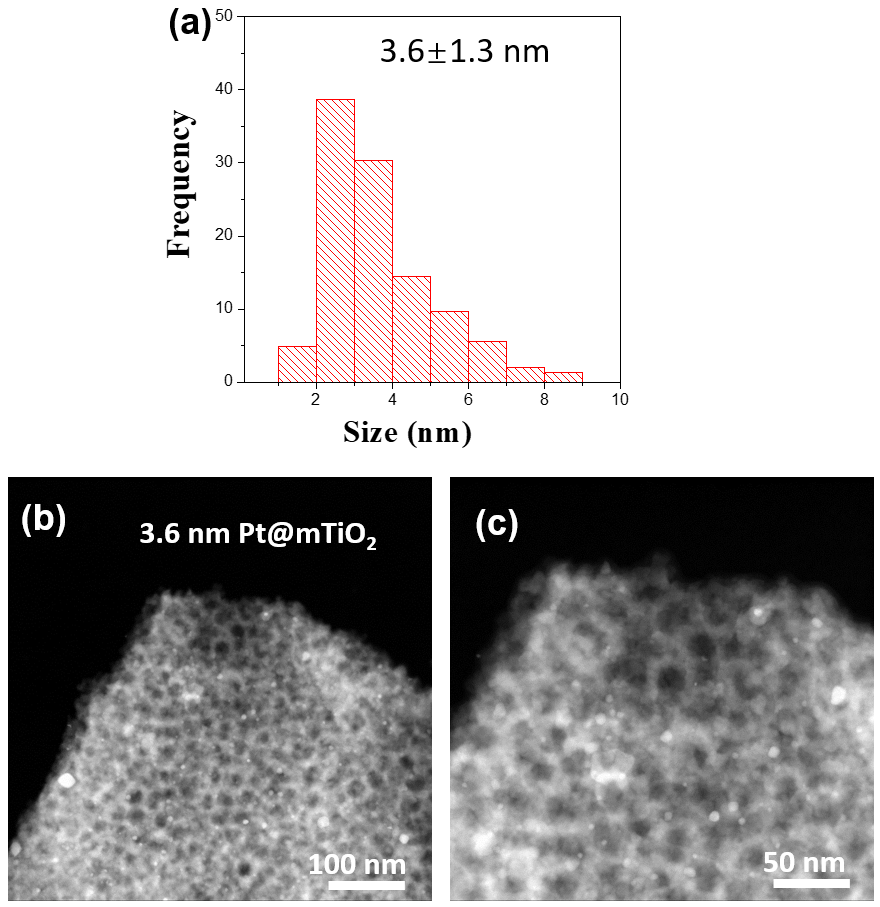


**Figure S8.** (a) The histogram of Pt particle-size distributions, (b,c) HAADF-STEM images of the 3.6 nm Pt on mTiO_2_ prepared after 650 ^o^C thermal calcination.


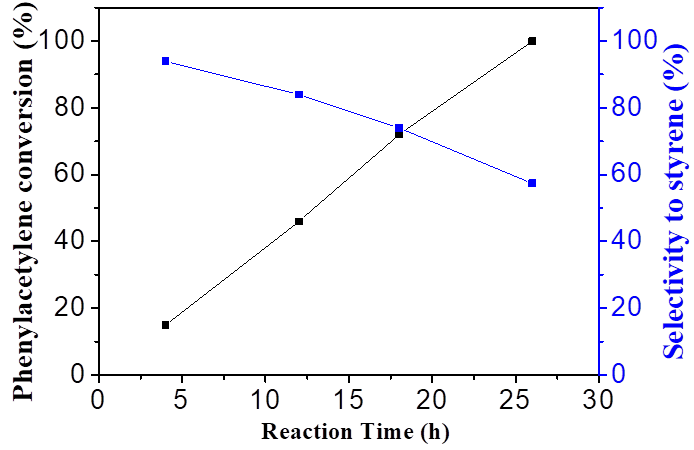


**Figure S9.** Phenylacetylene conversion and styrene selectivity of the 3.6 nm Pt NPs on mTiO_2_ as a function of reaction time.


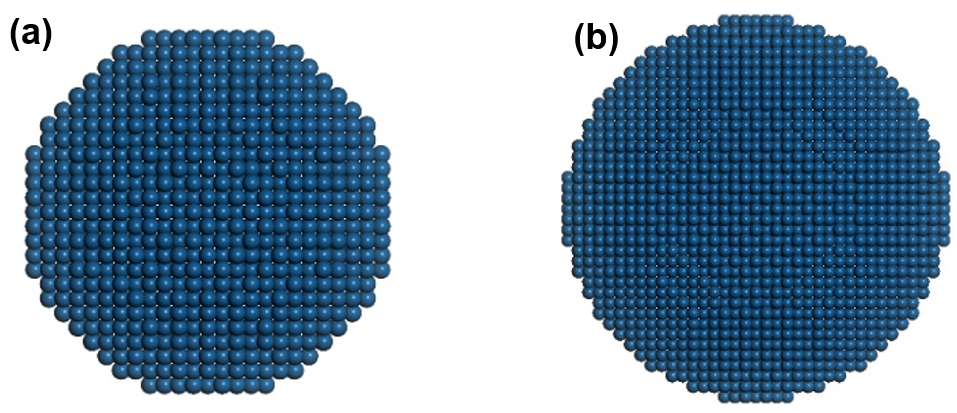


**Figure S10.** (a) Schematic model of the Pt NPs with a diameter of 2.5 nm. (b) Schematic model of the Pt NPs with a diameter of 3.6 nm.

**Table S1.** Comparison of initial Pt loading and that from EDS measurements in 2.5 nm Pt@mTiO_2_.

| **Catalyst** | **Pt loading from EDS** | **Initial Pt loading** |
| --- | --- | --- |
| **2.5 nm Pt@mTiO_2_** | **1%** | **1%** |

**Table S2.** The average cell parameters of the 2.5 nm Pt@TiO_2_ and mTiO_2_ calculated by three small-angle X-ray scattering (SAXS) reflections.

| **Crystal plane** | ***q* (nm^-1^)** | **a (nm)** |
| --- | --- | --- |
| **(111)** | **0.29** | **37** |
| **(311)** | **0.53** | **40** |
| **(500)** | **0.84** | **37** |

The calculations are according to the following equation, in which d is the d spacing, q is the scattering vector, (hkl) are the Miller indices and a is the cell parameter. The average cell parameter is calculated to be 38 nm by three small-angle X-ray scattering (SAXS) reflections.

$$d= \frac{2\pi}{q}$$

$$a^{2}=d^{2}(h^{2}+k^{2}+ l^{2})$$

**Table S3.** Comparisons of styrene selectivity at full phenylacetylene conversion of Pt@mTiO_2_ and previous results.

| Catalysts | Pd loading/% | | Phenylacetylene conversion/% | | Styrene  Selectivity/% | Reference |
| --- | --- | --- | --- | --- | --- | --- |
| Pt@mTiO_2_ | | 1 | | 100 | 80.3 | This paper |
| Pt/CNTs | | 0.79 | | 100 | 0 | Catal. Today. 2012, 186, 69 |
| Pt/γ-Al_2_O_3_ | | 0.5 | | 100 | 0 | Ind. Eng. Chem. Res. 2002, 41, 3345 |
| Lindlar Catalyst | | -- | | >99 | 60 | Adv. Mater. 2016, 28, 4747 |
| Pd/Al_2_O_3_ | | 5 | | >99 | 35 | Adv. Mater. 2016, 28, 4747 |
| PdHHDMA/TiS | | -- | | >99 | 50 | Adv. Mater. 2016, 28, 4747 |
| Pd NPs | | -- | | >99 | 0 | ACS Catal. 2019, 9, 4632 |
| Pd/CuS | | 2 | | 100 | 0 | Nano Res. 2016, 9. 1209 |
| Pd/MDPC | | -- | | 97 | 62 | ChemCatChem, 2016, 8,1111 |
